# Research Progress on Fumonisin B1 Contamination and Toxicity: A Review

**DOI:** 10.3390/molecules26175238

**Published:** 2021-08-29

**Authors:** Jia Chen, Jun Wen, Yating Tang, Jichao Shi, Guodong Mu, Rong Yan, Jing Cai, Miao Long

**Affiliations:** 1 College of Animal Science & Veterinary Medicine, Shenyang Agricultural University, Shenyang 110866, China; 2020200157@stu.syau.edu.cn (J.C.); 2020240593@stu.syau.edu.cn (J.W.); 2019220559@stu.syau.edu.cn (Y.T.); 2018220520@stu.syau.edu.cn (R.Y.); 2018200143@stu.syau.edu.cn (J.C.); 2 Liaoning Service Development Center, Shenyang 110032, China; sjc6319@126.com; 3 Jilin Center for Animal Disease Control and Prevention, 4510 Xi’an Road, Changchun 130062, China; dashaom@126.com

**Keywords:** fumonisin B1, toxicity, mechanism, contamination, review

## Abstract

Fumonisin B1 (FB1), belonging to the member of fumonisins, is one of the most toxic mycotoxins produced mainly by *Fusarium proliferatum* and *Fusarium verticillioide*. FB1 has caused extensive contamination worldwide, mainly in corn, rice, wheat, and their products, while it also poses a health risk and is toxic to animals and human. It has been shown to cause oxidative stress, endoplasmic reticulum stress, cellular autophagy, and apoptosis. This review focuses on the current stage of FB1 contamination, its toxic effects of acute toxicity, immunotoxicity, organ toxicity, and reproductive toxicity on animals and humans. The potential toxic mechanisms of FB1 are discussed. One of the main aims of the work is to provide a reliable reference strategy for understanding the occurrence and toxicity of FB1.

## 1. Introduction

**Figure 1:**
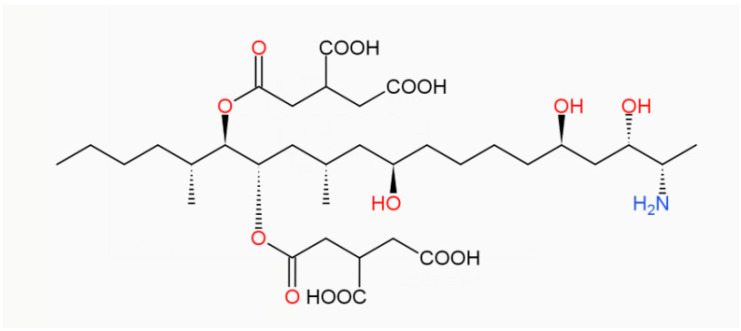
Chemical structure of FB1.

Fumonisins (FUMs) are mycotoxins produced from *Fusarium* spp. Among them, the amount of fumonisins produced by *Fusarium verticilliodes* and *Fusarium proliferatum* is the most pronounced [1,2]. In many countries, fumonisin B1 (FB1) is produced by *Gibberella fujikuroi* var. moniliformis (*Fusarium verticillioides*) [3,4]. It was first isolated from *Fusarium moniliforme* MRC 826 in 1988 by South African scientist Gelderblom et al. [5]. In the same year the structure of fumonisin was detected by mass spectrometry and 1H and 13C n.m.r [6]. The structure of fumonisins is similar to that of sphingolipids, with the main chain containing 19–20 carbon atoms and the rest of the various groups distributed on both sides of the main chain (Figure 1). So far, Fumonisins are classified into four major groups, FA, FB, FC, and FP, depending on the type of moiety. Among them, FB1, FB2, and FB3 are most frequently found in food and often occur together in a ratio of approximately 68:20:12 [7]. FB1 is the most abundant and toxic, posing the greatest threat to animal and human health, and most scholars are conducting research on FB1 [8]. Fumonisin B1 is a water-soluble mycotoxin that is stable to heat, and its chemical structure is shown in Figure 1. Animal ingestion of FB1-containing feed can cause a series of types of physical damage, such as pulmonary edema and hydrothorax in pigs, leukoencephalomalacia in horses, etc. Human consumption of foods containing FB1 may cause esophageal cancer, liver, and kidney disease. Women who are exposed to high levels of fumonisins in their diet during early pregnancy have a high risk of bearing a child with a birth defect of the brain or spinal cord [9,10]. FB1 also has the potential to cause heart failure in humans due to damages in myocardial contractility and massive blood influx, known as Idiopathic Congestive Cardiopathy (ICC) [11]. The aim of this study was to investigate the toxic effects of FB1 on animals and humans, as well as the toxic mechanism of FB1, and to provide a reference for subsequent research. 

## 2. Contamination Caused by FB1

FB1 is mainly formed by the fungi encountered in the growing crop prior to harvesting, unlike ochratoxins and aflatoxins, and therefore development of the same fungi is often difficult to control or prevent [12]. It mainly contaminates corn and corn products [13,14]. In addition, FB1 also contaminates rice and other cereals such as oat, rye, barley, wheat, but at lower levels (Table 1). In 66 samples of maize and rice from Golestan province, Iran, FB1 was present in 50% of maize samples and 40.9% of rice samples with average levels of 223.64 μg/g and 21.59 μg/g, respectively [15]. The European Community and the European Union have clear standards for the maximum limit of fumonisin in human foods and animal feeds (Table 2) [11]. Studies have shown that a hot and humid environment can lead to the increase of FB1 content [16,17,18]. Fumonisins showed high stability in Maize at temperatures from 28.97 to 32.14 °C, humidity from 27.29 to 32.14%, and pH from 5.5 [19,20]. Thus, the hazard of FB1 to agricultural crops is reported more in countries in temperate tropical regions.

Since the feed is composed of corn and other cereals, contamination of animal feed by FB1 should be brought to attention (Table 1). In Bulgarian pig farms/chicken farms, the average FB1 content in feed samples in 2006 and 2007 was 5564.1 + 584.4 μg/kg and 3254.5 ± 480.6 μg/kg [3]. In the feed samples from the South African pig farms, the mean FB1 levels detected in 2007 and 2008 were 5289 ± 1034 μg/kg and 5021 ± 844 μg/kg [4]. These farms are plagued by kidney problems. In such a high concentration of FB1, this disease is likely to involve FB1. In a Spanish survey of pig feed samples, FB1 was also detected at a maximum of 3959 μg/kg [21]. Pietsch conducted a risk assessment on FB1 in European fish feed. The sum of FB1 in the 12 feed components was 3576 μg/kg [22]. Contamination with FB1 has been reported in feed ingredients and compound feeds in Korea, and its content is high, reaching a maximum of 14,900 ± 72.6 ng/g [23]. In Chinese feed tests, 404 out of 420 feeds were contaminated, with the highest level being 6568 μg/kg [24]. This seriously endangers the herd health and economic development. In the pet industry, the main components of some pet foods are corn and wheat, so FB1 can also contaminate pet foods. Although it is not abundant, it also causes chronic damage to pet bodies [25].

The effect of FB1 on humans depends mainly on the dietary habits of the area where they live. People in areas where corn and corn products are regularly consumed are exposed to FB1. Wheat products, although FB1 is also present but not at high levels, are less harmful to humans. In three remote Tanzanian villages, in which the child’s food during months 12–22 was predominantly corn based, 96% of urine samples tested positive for urinary fumonisin B1(UFB1) [26]. Kigwa had the highest UFB1 geometric mean 327.2 (217.1–493.0) pg/mL, followed by Nyabula at 211.7 (161.1–278.1) pg/mL, while Kikelelwa had the lowest at 82.8 (58.3–117.7) pg/mL [26]. This was attributed to the different corn intakes in the three villages. Another experiment in Tanzania also demonstrated in three regions that the effect of FB1 on humans is related to corn intake, and its exposure to FB (FB1 + FB2) in 12-month-old infants was 4 μg/kg body weight/day [27]. This value is higher than the provisional maximum tolerable daily intake (PMTDI) set by JECFA (2 µg/kg BW per day) [7]. Besides that, there are many countries whose exposure levels also exceed the PMTDI, such as Algerian, China, Mexico, etc. (Table 3). In the Netherlands, in people with celiac disease gluten induced enteropathy (intolerance to foods containing wheat gum) who were regularly exposed to corn, 37% of them consumed at least 105 ng FB1 and 97% of them at least 103 ng FB1 per day [28]. It could also prove that the effects of FB1 in humans are mainly dependent on the amount of corn consumed. In Brazil, corn is cheap, so industrial beer with corn as the main ingredient is also contaminated by FB1 [29]. Various corn foods can also be contaminated by FB1, such as popcorn, cornmeal, corn snack, etc. (Table 1).

**Table 1:** Contamination of agricultural crops, human food, and animal feed by FB1.

Commodity	Country	Positives/Total	Content	Reference
**Agricultural products**				
Maize	South Africa (Limpopo Province)	49/54	101–53,863 μg/kg	[30]
Maize	Algeria	29/30	289–42,143 μg/kg	[31]
Rise	Ecuador (Guayas)	3/20	22.6–54.3 μg/kg	[32]
Rise	Ecuador (Los Ríos)	7/23	17.9–1146.4 μg/kg	[32]
Maize grains	Iran	56/82	15/155 μg/kg	[33]
Cereal mixtures	Brazil	99/105	137.8 ± 257.4 μg/g (Mean)	[34]
Corn samples (moldy in 1993–1995)	Hungary	51/69	0.05–75.1 mg/kg	[35]
Corn samples (mold-free in 1994)	Hungary	7/23	0.06–5.1 mg/kg	[35]
Maize	Spain	48/55	0.2–19.2 μg/g	[36]
Barley	Spain	21/29	0.2–11.6 μg/g	[36]
Wheat	Spain	8/17	0.2–8.8 μg/g	[36]
Soybeans	Spain	1/1	8.7 μg/g	[36]
Oats	Spain	29/100	62.3–217.4 μg/kg	[37]
Maize	China	166/249	530–10,315 μg/kg	[38]
Corn	China (Huantai)	28/30	nd–12.5 mg/kg	[39]
Rice	China (Huantai)	8/9	nd–0.4 mg/kg	[39]
Wheat flour	China (Huantai)	8/9	nd–0.4 mg/kg	[39]
Corn	China (Huaian)	43/43	0.2–5.9 mg/kg	[39]
Rice	China (Huaian)	9/10	nd–0.3 mg/kg	[39]
Wheat flour	China (Huaian)	5/7	nd–0.4 mg/kg	[39]
Corn	China (Fusui)	29/34	nd–37.0 mg/kg	[39]
Rice	China (Fusui)	9/10	nd–0.5 mg/kg	[39]
**Human food products**				
Corn grits	Brazil	2/2	0.17–1.23 μg/g	[40]
Corn meal	Brazil	9/9	0.56–4.93 μg/g	[40]
Degerminated corn	Brazil	8/11	nd–4.52 μg/g	[40]
Popcorn	Brazil	4/9	nd–1.72 μg/g	[40]
Precooked corn flour	Brazil	4/6	nd–1.79 μg/g	[40]
Industrial beers	Brazil	56/114	201–1568 μg/L	[41]
Cornmeal	Brazil	25/32	33–1208 μg/kg	[42]
Corn-flour	Brazil	19/25	114.4–558.6 μg/kg	[42]
Popcorn	Brazil	32/39	102.0–1127.3 μg/kg	[42]
Polenta	Brazil	2/2	149.0–214.2 μg/kg	[42]
Breakfast cereals (corn-based)	Canada	30/34	nd–1980 μg/kg	[43]
Breakfast cereals (oat-based	Canada	5/19	nd–57 μg/kg	[43]
Breakfast cereals (rice-based)	Canada	2/29	nd–5 μg/kg	[43]
Breakfast cereals (wheat-based)	Canada	5/29	nd–51 μg/kg	[43]
Broa (typical Portuguese maize bread)	Portugal	24/80	nd–448 μg/kg	[44]
Cornmeal	Portugal	41/41	50–1300 μg/kg	[45]
Sweet corn	Portugal	36/41	50–400 μg/kg	[45]
Popcorn grain	Japan	49/57	67.5 μg/kg (Mean) 354 μg/kg (Maximum)	[46]
Corn grits	Japan	46/46	104 μg/kg (Mean) 1380 μg/kg (Maximum)	[46]
Corn snacks	Japan	41/50	113 μg/kg (Mean) 1670 μg/kg (Maximum)	[46]
**Animal feeds**				
Feed samples (2007)	South African	20/24	5289 ± 1034 μg/kg (Mean)	[4]
Feed samples (2008)	South African	19/24	5021 ± 844 μg/kg (Mean)	[4]
Feed samples (2006)	Bulgarian	24/25	5564.1 ± 584.4 μg/kg (Mean)	[3]
Feed samples (2007)	Bulgarian	23/25	3254.5 ± 480.6 μg/kg (Mean)	[3]
Compound feedstuff	China	284/300	20–6568 μg/kg	[24]
Concentrated feedstuff	China	60/60	23–6239 μg/kg	[24]
Premixing feedstuff	China	60/60	341–6004 μg/kg	[24]
Cattle feeds (breeding)	Korea	10/10	365 ± 6.23–13,900 ± 179 ng/g	[23]
Cattle feeds (lactation)	Korea	8/8	411 ± 149–2160 ± 471 ng/g	[23]
Cattle feeds (fattening)	Korea	32/32	430 ± 173–13,300 ± 2070 ng/g	[23]
Swine feeds (breeding)	Korea	30/42	363 ± 142–14,900 ± 72.6 ng/g	[23]
Swine feeds (fattening)	Korea	8/8	1510 ± 174–14,600 ± 120 ng/g	[23]
Poultry feeds (layer)	Korea	22/24	73.2 ± 15.4–12,800 ± 1460 ng/g	[23]
Poultry feeds (broiler)	Korea	17/22	1380 ± 169–14,600 ± 187 ng/g	[23]
Cat food samples (maize)	Poland	-	10.0–15.6	[25]
Cat food samples (maize and wheat)	Poland	-	15.0–20.8	[25]
Dog food samples (maize)	Poland	-	29.5–55.5	[25]
Dog food samples (maize and wheat)	Poland	-	26.5–57.0	[25]
Dog food samples (wheat)	Poland	-	29.6–37.1	[25]

**Table 2:** EC and EU maximum limits for fumonisins (FB1 + FB2) in human foods and animal feed.

	Human Foods and Animal Feeds	Maximum Levels (mg/kg)
1	**Human foods**	
2	Unprocessed maize, with the exception of unprocessed maize intended to be processed by wet milling	4
3	Maize intended for direct human consumption, maize- based foods for direct human consumption, with the exception of foodstuffs listed in 3 and 4	1
4	Maize-based breakfast cereals and maize-based snacks	0.8
1	Processed maize-based foods and baby foods for infants and young children	0.2
	**Animal feeds**	
	Maize by-products	60
	Complete and complimentary feedstuffs for pigs, Equidae, rabbits	5
	Complete and complimentary feedstuffs for poultry, calves, lambs, kids	20
	Complete and complimentary feedstuffs for adult ruminants and mink	50
	Complete and complimentary feedstuffs for fish	10

**Table 3:** FB1 exposure levels in people from different countries.

Country	Ingestion Population	Exposure Levels	Reference
South Korea	[50.2 ng/g × 0.1 g per person per day]/57.6 kg	0.087 ng/kg of body weight per day	[47]
China (Huantai)	-	92.4 μg per day	[39]
China (Huaian)	-	460.0 μg per day	[39]
China (Fusui)		138.6 μg per day	[39]
China (Shandong Province)	-	0.02 µg/kg bw/day	[48]
Algerian	-	10.86 µg/kg bw/day	[31]
Brazil (rural areas with higher corn intake)	A 70 kg adult	1276 ng/kg bw/day	[40]
Brazil (urban and some rural areas)	A 70 kg adult	392 ng/kg bw/day	[40]
Tanzania	Children aged 6–12 months	2 μg/kg body weight/day	[27]
Iran (Isfahan 1998)	3.3 g maize/person (60 kg)/day	0.009 μg/kg b.w./day	[49]
Iran (Isfahan 1998)	3.3 g maize/person (60 kg)/day	0.012 μg/kg b.w./day	[49]
Iran (Mazandaran 1998)	3.3 g maize/person (60 kg)/day	0.125 μg/kg b.w./day	[49]
Iran (Mazandaran 1998)	3.3 g maize/person (60 kg)/day	0.175 μg/kg b.w./day	[49]
Iran (Mazandaran 1998)	3.3 g maize/person (60 kg)/day	0.338 μg/kg b.w./day	[49]
Mexico	For men of 73.3 kg bw	4.12 µg/kg bw/day	[50]
Mexico	For women of 65.8 kg bw	3.00 µg/kg bw/day	[50]
Hungary	All maize-product consumers	0.045–0.120 µg/kg bw/day	[51]

## 3. Toxic Mechanism of FB1

### 3.1. Effects on Sphingolipids

FB1 is a kind of diesters composed of different polyhydric alcohols and propionic acids. Its structure is composed of a long hydroxylated hydrocarbon chain, as well as surrounding tricarboxylic acids, methyl, and amino groups, and it resembles that of sphingolipids [52]. Sphingolipids are components of the plasma membrane which are regulators of cell-to-cell interactions and cell-to-cell recognition [53]. The hydrolytic skeleton of FB1 is very similar to sphingosine bases of sphingolipids (sphinganine (Sa) and sphingosine (So)). Sa is condensed by serine palmitoyl COA in vivo. Sa and So synthesize ceramide (Cer) under the action of ceramide synthase. FB1 can compete with Sa and So for ceramide synthase, inhibit ceramide synthase, leading to the production of dihydroceramide ceramide. In addition, the synthesis of sphingolipid complex (SLS) was decreased, the amounts of Sa and So increased, and the contents of sphinganine-1-phosphate and sphingosine-1-phosphate increased. It has been shown that the elevation of these substances is present before a significant rise in liver enzymes, so Sa, So, and Sa/So can be used as markers of FB1 exposure and thus detect FB1 exposure [54,55,56]. According to the study, intravenous and oral administration of FB1 (exposure levels of 139 nmol and 3425 nmol b.w., respectively) resulted in a significant increase in the Sa/So ratio in the blood and cerebrospinal fluid of pigs, while treatment with fumonisin esterase resulted in a similar Sa/So as in the control group, suggesting that FB1 did interfere with sphingolipid metabolism [52]. Generally, the Sa is elevated early in the process and the elevation is large: So is elevated later and the elevation is smaller [57]. The ratios of Sa and So are time- and dose-dependent, and can vary depending on the amount of FB1 residing in the organ, which is related to the cell-specific function of sphingolipids in the same organ of different species.

### 3.2. Oxidative Stress

Some studies have found that FB1 intake also causes oxidative stress (OS). OS is a state of imbalance between reactive oxygen species (ROS) formation and antioxidant defense mechanisms in the body, and many diseases of old age are directly related to OS [58]. When animals ingest FB1, intracellular markers of OS, such as ROS, are increased and generate a highly-oxidized environment with an increased content of the intracellular antioxidant [59]. When ROS concentration exceeds antioxidant capacity, OS occurs in cells [60].

Oxidative stress of FB1 induced cytotoxicity and decreased cellular activity. In the study, it was found that FB1 (100 mM for 0–144 h) induced a dose-dependent increase of ROS production in C6 glioblastoma and GT1-7 hypothalamic cells, but had no significant effect on SH-SY5Y cells [61]. In parallel, these three cell lines exhibited decreased glutathione (GSH) levels, increased malondialdehyde (MDA) production, lipid peroxidation and necrotic cell death [61]. Pig Iliac Endothelium Cells (PIECs) were treated with 50 μg/mL FB1, and the content of MDA increased and GSH decreased after 48 h. At the same time, the activities of antioxidant enzymes such as Superoxide dismutase (SOD), Glutathione peroxidase (GSH-Px), Catalase (CAT), and Thioredoxin reductase (TrxR) decreased significantly, and the elevation of MDA leads to cell membrane lipid peroxidation, resulting in cell membrane damage and reduced cell activity. The barrier functional integrity of PIECs cells was also measured, and it was found that FB1 may affect the expression of tight junction proteins in porcine vascular endothelial cells and alter the intercellular junctions [62].

The oxidative stress caused by FB1 induces DNA damage, and DNA damage induces cell carcinogenesis. It has been shown that FB1 increases the level of 8-hydroxy-2′-deoxyguanosine (8-OH-DG) in rat C6 glioma cells and p53-null mouse embryo fibroblasts [63]. 8-OH-DG is a biomarker of oxidative DNA damage, which induces DNA hypomethylation and thus affects cellular stability [64]. This result is identical to the study by Demirel et al. and Arumugam et al. [65,66]. In the experiments on rat liver epithelial cell line (Clone 9) and rat kidney proximal tubular epithelial cell line (NRK-52E), FB1 leads to methylation of tumor-related genes (p16, VHL, E-cadherin, and c-myc) [65]. Arumugam et al. showed that, in HepG2 cells, FB1 suppressed the activity of checkpoint kinase 1 (CHK1) to generate DNA damage by regulating phosphatase and tensin homolog (PTEN), phosphoinositide 3-kinases (PI3K) and protein kinase B (Akt) [66]. This result is similar to that of Yu et al. [67]. They found that FB1 was through the PI3K/Akt signaling pathway, which regulated cell survival. They also found that FB1 could increase the expression of HDACs in HEECs [67]. Among epigenetic proteins, histone deacetylases (HDACs) have emerged as potential therapeutic targets for many diseases including cancer. Aberrant epigenetic changes may lead to chromatin instability and reduced levels of histone acetylation, which may be responsible for FB1 carcinogenesis. FB1 also causes methylation of RNA, which is due to elevation of N6 methyladenosine (m6A). Meanwhile, hypermethylation of the kelch-like ECH associated protein 1 (Keap1) promoter and hypomethylation of the related factor 2 (Nrf2) promoter, a decrease in mir-27b, and an increase in m6a-keap1 and m6a-nrf2 also lead to the activation of the keap1-nrf2 signaling pathway [68].

Oxidative stress of FB1 induces apoptosis and cellular autophagy. ROS production in cells downregulates MAPKs-specific phosphatases, thereby affecting the activation of mitogen-activated protein kinase (MAPKs) [69]. FB1 at 0.5 mg/kg was administered to the liver and kidney of rats, and the signal of c-Jun N-terminal kinase (JNK) was detected in the liver after 7 days, and the signal of all three MAPKs (extracellular regulated protein kinases (ERK), JNK, and P38) was increased in the kidney [70]. It was also reported that FB1 could significantly increase the phosphorylation of JNK protein, but had no obvious effect on the phosphorylation of ERK and p38 [71]. In a mouse colon model, FB1-mediated oxidative stress and Ca++ release from the endoplasmic reticulum induced JNK phosphorylation, activated P53 signaling, led to the expression of apoptotic signaling molecules Programmed Cell Death (PUMA) and Caspase-3, and induced apoptosis [70]. BCL2-Associated X (Bax) is a pro-apoptotic gene, which is activated by P53 in vivo and enters the mitochondria to help produce cytochrome C, which further produces Caspase-9 and activates Caspase-3 to induce apoptosis [72]. Oxidative stress-mediated activation of JNK simultaneously leads to phosphorylation of B-cell lymphoma-2 (Bcl-2) and release of Beclin1, which indirectly stimulates the expression of LC3- II or LC3- I and induces autophagy [70].

### 3.3. Endoplasmic Reticulum Stress

When cells are strongly stimulated by various factors, the ability to correctly fold and post-translationally modify the secreted transmembrane proteins in the endoplasmic reticulum is hindered, resulting in a large accumulation of misfolded proteins in the organelles and causing endoplasmic reticulum stress [73].

FB1 induces ER stress in mouse liver cells and HepG2 cells. After treating mouse hepatocytes and HepG2 cells with FB1, we found that the expression of protein kinase R-like ER kinase (PERK), Inositol-requiring enzyme-1αIRE1-α, and autophagy marker (LC3I/II), which are related to autophagy in ER stress, were significantly increased. The phosphorylation expression of AMP-dependent protein kinase (AMPK) is elevated, and the phosphorylation expression of mammalian target of rapamycin (mTOR) is decreased, thereby triggering cell autophagy [39]. This notion was also demonstrated by Hou et al., who found that FB1 was through mTORC1 to mediate autophagy induced nephrotoxicity [74]. The expression level of glucose regulatory protein 78 (Bip), activated transcription factor 4 (ATF4) and C/EBP homologous protein (CHOP) was also significantly elevated in HepG2 cells [75]. It has been suggested that this is due to the activation of the PERK-CHOP signaling pathway by FB1, which induces apoptosis [76]. The toxic effect of endoplasmic reticulum on AML12 mouse liver cells was reported to be through the IRE1α pathway, but not the PERK pathway, but the mechanism of the IRE1α signaling pathway was not described and may require further study [77]. Meanwhile, two experiments in GES-1 and AML12 mouse liver cells suggest that the endoplasmic reticulum stress pathways caused by different organs may be different, and the specificity to organs needs to be further investigated. This report also showed that inhibition of endoplasmic reticulum stress significantly reduced the hepatotoxicity of FB1, which may be a good way to reduce the toxicity of FB1 to the liver [77].

### 3.4. TNF Signaling Pathway

The toxicity of FB1 is related to TNF-α [78]. In experiments with Gastric epithelial (AGS) and human colon adenocarcinoma cell line (SW742), FB1 was found to induce a dose-dependent increase in the expression of tumor necrosis factor (TNFα) and IL-1β in these two cell types, thus suggesting that FB1 can promote cytokine production by gastrointestinal cells, which may underlie the subsequent onset of inflammation [79]. Recent articles have shown that FB1 can upregulate the expression of TNF signaling pathway-related mRNA in porcine kidney cells (PK-15) cells and that tumor necrosis factor α (TNFα) is a key substance causing toxicity [80]. It was also demonstrated that NF-κB is an important target in the TNF signaling pathway [80]. The involvement of this signaling pathway was also found in porcine jejunum and liver, where a significantly elevated expression of NF-κB and Interleukin-8 (IL-8) could be detected, leading to apoptosis [81]. 

**Figure 2:**
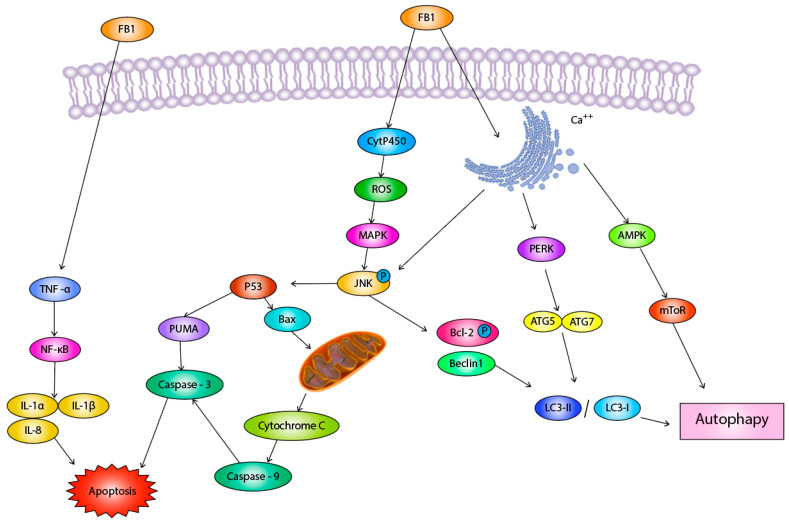
Mechanism of FB1 toxicity.

The mechanism diagram of FB1 is shown in Figure 2. 

## 4. Toxic Effects of FB1

### 4.1. Immunotoxicity

FB1 has some immunotoxicity. Intragastric administration of FB1 (80 mg/kg BW) to mice for two weeks reduced spleen weight and also caused 12.9% thymocyte apoptosis [82]. This finding is akin to that of avian species, where FB1 significantly reduced splenocyte activity at a dose of 50 μg/mL and the results were consistent at 24, 48, and 72 h [83].

FB1 acted on cytokine expression, and one study showed that gavage of BALB/c mice with FB1 (100 mg/kg) for two weeks resulted in an increased expression of interleukin-10 (IL-10) and interleukin-4 (IL-4) mRNA in the spleen and a decreased expression of interferon-γ (IFN-γ) and tumor necrosis factor (TNFα) mRNA [82]. Interestingly, Taranu et al. (2005) found that the exposure of weaned piglets to 1.5 mg/kg could decrease IL-4mRNA expression and increase IFN-γ synthesis, which may be related to species and FB1 dose. The FB1 uptake was found to interfere with the specific immune response of animals during vaccination. This is because FB1 decreases the level of specific antibodies in the serum of piglets, thus causing a decrease in the specific immune response to the vaccine antigen [84]. This is similar to the findings of Stoev et al. [85]. He found that when the content of FB1 in the feed was 10 mg/kg, it could significantly reduce the antibody titer and interfere with the humoral immune response during vaccination. In experiments by Li et al. (2017), it was found that FB1 reduced the immune responsiveness of bone marrow-derived dendritic cells (BMDCs), and the number of dendrites in BMDCs was significantly decreased under treatment using 1000 ng/mL FB1 compared to positive controls, and LPS-induced expression of CD80, CD86, and MHCII changed from up-regulation to down-regulation in response to FB1 [86]. In humans, FB1 inhibits the expression of HLA class I antigen and low molecular weight proteasome 2 (LMP2) and transporter associated with antigen presentation (TAP1) and increases the chance of survival of abnormal cells [87]. This is similar to the results of aflatoxin G1 [88]. This result may induce cancer, but its relationship with cancer needs further experimental study [87]. However, FB1 had no effect on HLA-C expression at the mRNA level [87]. 

Immune cells were important targets for the toxic effects of FB1 [89]. After 72 h of exposure at a concentration of 101.15 μg/mL, the cellular activity of porcine lymphocytes decreased to 50% of its original level [90]. Lymphocyte survival in humans decreased by 3.5% and 11.3% after 24 h of exposure to concentrations of 5 and 20 μg/mL, respectively [91]. Macrophage chemotaxis and phagocytosis were reduced when FB1 (15 mg/kg) was administered to broiler chickens [92]. A decrease in macrophage capacity led to metabolic and immune system disorders in birds, intensifying the severity of chlamydia [93]. FB1 can also suppress the non-specific immune system of pigs, reducing macrophage capacity and exacerbating pathogen infection. A study of the effects of FB1 on Mycoplasma pneumoniae infection in swine found that feeding FB1 at 20 mg/kg could aggravate the progression of infection [94]. FB1 also significantly increases colonization of the small and large intestine by extra-intestinal pathogenic E. coli strains [95]. Some studies have shown that administration of different doses of FB1 (11.8 mg/kg, 0.5 mg/kg, and 20 mg/kg) can complicate the infection process of Bacillus subtilis A, causing interstitial bronchial pneumonia [96,97,98]. Recent studies have shown that non cytotoxic doses of FB1 can aggravate OTA induced cytotoxicity and apoptosis. This process has JNK/MAPK pathway involvement [71]. There are currently many human and animal foods that still contain high doses of FB1.Therefore, the synergistic mechanism of FB1 against various pathogenic flora and microorganisms should be further investigated, so as to evaluate the extent to which FB1 is involved in the development of infectious diseases in humans and animals.

### 4.2. Organ Toxicity

A series of studies have shown that FB1 has certain toxic effects in organs such as liver, lung, kidney, heart, and intestine in different animals (Table 4). After continuous gavage delivering FB1 (100 mg/each) for 5 to 11 days, samples of various organs were collected and analyzed for FB1 residues: particularly high levels were detected in kidney (mean 824 mg/g), liver (231 mg/g), lung (170 µg/g), and spleen (854 µg/g) [99]. A single subcutaneous injection of FB1 (25 mg/kg) in mice resulted in a sustained increase in Sa content in the kidney and in concentrations much greater than those in the liver and intestine [100]. It has also been shown that FB1 intraperitoneal injection in C57BL6 mice (8 mg/kg BW) decreased oxidative defense in the liver and kidneys, while having no effect on the lungs [101]. In poultry, the highest concentration of FB1 was found in the liver, lower in the kidney than in the liver, and the muscles were the least contaminated [102]. This suggests that FB1 acts to different degrees in different organs of different animals. It has been suggested that this difference may be due to the ratio of ceramide CerS (CerS4, CerS2, and CerS1) protein expression in each tissue and the affinity of FB1 for these proteins [103]. Among them, the liver and kidney are important targets for the toxic effects of FB1 on the organism.

#### 4.2.1. Toxic Effects of FB1 on the Liver

The liver is one of the main target organs of FB1 in the body, and the main symptoms are cirrhosis, failure, and in severe cases, liver necrosis and liver cancer. Treatment of rats by gavage (FB1, 50 mg/kg, 6 doses over 11 days) induced hepatic edema, intrahepatic monocyte necrosis, and also caused early phenomena of lipid accumulation and liver fibrosis [104]. In a pig trial, after feeding FB1 (1.5 mg/kg BW) for nine consecutive days, liver weight did not increase, but plasma total cholesterol (TC) and aspartate transferase (AST) were significantly elevated, indicating liver damage [103]. After feeding feeds with FB1 (7.5 mg/kg and 10 mg/kg for 196 days) to rabbits, hepatic necrosis developed and a large number of macrophages and lymphocytes were found in the periphery [105]. For this phenomenon, Neelesh et al. performed experiments with mice and concluded that the production of pro-inflammatory cytokines after T-cell activation is an important mechanism by which FB1 causes hepatotoxicity in mice and that this mechanism does not affect the accumulation of sphingoid bases [106].

FB1 may induce liver cancer. FB1 induced liver tumors in female B6C3F1 mice [107]. In rats treated by gavage it was shown that the effective dosage level (EDL) for cancer over 21 days was 14.2 < EDL < 30.8 mg Fb1/100 g bw, and the EDL value for carcinogenicity within 14 days was 23.3 < EDL < 33.3 mg Fb1/100g bw [104]. It has been shown that FB1 induces cancer by regulating the levels of fatty acids, cholesterol, and sphingolipids, leading to the disruption of membrane microdomains and lipid rafts [108]. It has also been shown that FB1 causes methylation of oncogene (c-myc) in the rat liver epithelial cell line (Clone 9), suggesting that methylation of DNA is also a cause of cancer [65].

#### 4.2.2. Toxic Effects of FB1 on the Kidney

The kidneys are also highly sensitive to FB1. It has been shown that the concentration of FB1 that caused nephrotoxicity in Sprague-Dawley rats in a 4-week feeding study was much lower than that required to cause hepatotoxicity [109]. This was also demonstrated by Szabó et al. with a higher sensitivity of rat kidneys to FB1 at low doses and relatively short-term FB1 exposure [110]. Bondy et al. injected rats with purified FB1 (0.75 mg/kg) for six consecutive days and observed a small amount of renal damage and renal epithelial cell shedding [111]. This result is similar to that of Szabó et al. where the same shedding of renal epithelial cells and an increase in urine volume and potassium excretion were found after the administration of FB1 (≥50 mg/kg) to rats [112]. Renal epithelial cell shedding may lead to cell loss and impaired replacement, which may induce cancer [113]. Some characteristic changes can also be observed in the kidneys of pigs. Such as mild to moderate vacuolar or granular degeneration of proximal tubule epithelial cells, hyperaemia of vessels and peritubular capillaries, mild activation of capillary endothelial cells, mild mononuclear proliferation in interstitium, perivascular or pericapillary edema, and enlarged lymphatic vascular spaces. Protein debris is seen within the lumen of some renal tubules [85].

FB1 similarly induced renal tumors. The induction rate of renal tubular carcinoma was significantly increased in male F344 rats after feeding diets containing FB1 (50 mg/kg and 100 mg/km) [107]. The mechanism of FB1-induced renal tumors is similar to that in the liver. In rat kidney proximal tubular epithelial cell line (NRK-52E), promoter methylation of the tumor suppressor gene (VLH) was found, and FB1-induced DNA methylation was suggested to be an important cause of cancer induction [65]. It has also been found in NKR-52e that FB1 perturbs epigenetics by altering global histone modifications, which in turn induces cancer [114]. Further studies can provide more insight into the mechanism of FB1 toxicity.

#### 4.2.3. Toxic Effects of FB1 on the Intestinal Tract

FB1 causes disruption of the intestinal epithelial barrier, epithelial cell detachment, inflammatory cell infiltration, atrophy and necrosis, and different intestinal targets in different animals. In tests in pigs, oral administration of FB1-rich extracts was found to significantly increase the concentration of stress proteins in the colon, a response that was stronger than in the stomach or in the small intestine [115]. In contrast, in studies in rats, the damage was more severe in the duodenum and cecum [116], and the cause of this intestine-specific damage is not known. FB1 enhances the permeability of porcine intestinal epithelial cells (IPEC-J2), and long-term exposure to FB1 inhibits the proliferation of IPEC-J2, leading to an impaired cell barrier, a toxic effect that may be related to the ERK1/2 phosphorylation pathway [117]. This study also showed that the toxic effects of FB1 on the intestine were associated with elevated Ca2+ ions and alterations in cytochrome (CYP)-related enzymes [116].

#### 4.2.4. Toxic Effects of FB1 on the Heart and Lungs

The lung effects of FB1 were mainly caused by pulmonary edema in animals, with FB1 having the most serious effect on the lungs of pigs. The main pathological manifestations were the accumulation of serous or serofibrinous exdate in interlobular tissue or alveolar lumina and the thickening of interalveolar septa due to epithelial hyperplasia and/or accumulation of fibrin [85]. This is due to the cardiovascular effects induced by FB1 that block sphingosine mediated l-type Ca (2+) channels, which leads to left heart failure and triggers pulmonary edema [118]. FB1 decreases heart rate, myocardial contractility, and arterial blood pressure [119]. Alterations in the sphingolipid biosynthetic pathway affect the cardiovascular system of pigs. Elevated concentrations of sphinganine (Sa) and sphingosine (So) cause systemic hypotension, and elevated concentrations of sphingosine-1-phosphate cause pulmonary hypertension [120,121].

**Table 4:** FB1 organotoxic effects in various animals.

Animal Species	Method of Administration and Dosage	Duration	Effects	References
Mammals				
Holstein calf	Intravenous. 1mg/kg b.w.	4 days	Elevated sphingol and sphingosine concentrations in the liver, severe liver and bile duct damage, impaired liver function, apoptosis of liver cells.	[122]
Holstein calves	Mixed into the feed and fed 2.36 mg/kg bw, increasing to 3.54 mg/kg bw after 23 weeks	239~253 days	There was karyomegaly of hepatocellular nuclei, with occasional dense, shrunken hepatocyte nuclei and mitotic figures of hepatocytes. Billiary epithelial cells exhibited mild anisokaryosis and piling on of the epithelium.	[123]
Pigs	Mixed into the feed and fed. 20 mg/kg b.w	10 days	Relative increase in liver weight and vacuolar or fatty degeneration in hepatocytes.	[124]
Pigs	Mixed into the feed and fed. 10 mg/kg b.w	3 months	Degenerative changes in proximal tubules, hyperaemia of vessels and peritubular capillaries, activation of capillary endothelium, mononuclear proliferation in the kidney interstitium, perivascular or pericapillary edema in kidneys, etc.	[85]
Pigs	Intravenous. 1 mg/kg b.w	4 days	Mild pulmonary edema was present. In the liver, there was scattered hepatocyte apoptotic cell death and mitosis.	[125]
Piglets	Mixed into the feed and fed. 92 mg/kg b.w.	4~7 days	Fatal pulmonary edema.	[126]
Rats	Feed. 30 mg/kg b.w.	7 days	Pulmonary congestion, alveolar edema.	[127]
Rats	Feed. 50 mg/kg or 150 mg/kg	2 years	There was evidence of sustained nephrotoxicity manifested as basophilia, apoptosis, cell regeneration, and simple tubule hyperplasia, affecting proximal convoluted tubules in the deep cortex, extending into the outer region of the outer stripe of outer medulla.	[128]
Rats	Mixed into the feed and fed. 5 mg/kg	42 days	FB1 caused histological alterations in duodenum, cecum, and intestine, including partial shedding of villous epithelial cells and inflammatory cell infiltration.	[116]
F344/N/Nctr Br rats	Mixed into the feed and fed. 484 mg/kg	28 days	Induction of apoptosis and mitosis of hepatocytes in female rats. Induced apoptosis and regeneration of tubular epithelial cells in male rats.	[129]
Horses	Intravenous.0.2 mg/kg b.w.	7~28 days	Symptoms such as cyanosis, dyspnea and oedema of the mucous membranes and mild pulmonary oedema.	[130]
Male New Zealand rabbit	Feed, 1.5 mg/kg b.w.	21 days	Liver and kidney congestion with moderate vacuolar degeneration of the liver.	[131]
Poultry				
Japanese quail	200 mg/kg FB1and *Fusarium fujikuroi* culture material (MCM), supplying 100 mg/kg M	28 days	Cardiomyocytes thin and form many irregularly sized fluid vesicles between the myoplasm and myogenic fibers.	[132]

#### 4.2.5. Toxic Effects of FB1 on the Brain

FB1 can also cause damage to the brain, the most obvious example of which is leukoencephalomalacia in horses, which leads to neurotoxic symptoms and, in some cases, abnormal optic nerve function. The horse was given FB1 (0.2 mg/kg) daily, and over a period of four to 10 days, ataxia of the hindlimbs and trunk, depression, hypertension, and intermittent dementia developed. It has been suggested that this is because the intracranial pressure in horses is affected by eating with the head down and that FUMs block normal vascular regulatory mechanisms leading to cerebral edema [133]. Rats receiving FB1 (6.2 mg/kg) had a significantly reduced nerve conduction velocity after two weeks [134]. After pigs were fed a mold diet containing FB1 (10 mg/kg), the cerebral cortex was vacuolated and some neuronal and glial cytolysis under the meninges could be seen. The meninges showed signs of edema and congestion [85]. The necropsy of beef cattle having a sudden onset of blindness after grazing on standing corn contaminated with Fumonisin-producing Fusarium species revealed optic nerve degeneration and acute myelin edema [135]. Kovacić et al. added FB1 (100 mg/kg) to carp feed and found that FB1 can penetrate the blood-brain barrier of carp, causing degeneration and necrosis of nerve cells and brain edema [136]. FB1 has also been found to cause human embryonic neural tube defects (NTD) that inhibit neurodevelopment [137]. This phenomenon has also been observed in mouse models, where pregnant LM/BC mice injected with FB1 (20 mg/kg b.w.) at gestation day (GD) 7.5 and GD 8.5 had a 79% probability of developing NTD, and NTD was found to be preventable with timely folic acid supplementation [9]. However, Voss et al. experiment showed that folic acid deficiency did not aggravate the NTD (A29.75) induction in LM/BC mice [138]. At present, there is no clear experiment to show the mechanism of NTD induced by FB1, which needs further study.

#### 4.2.6. Toxic Effects of FB1 on Human Organs

FB1 may induce human esophageal cancer, which has been reported in Kenya, Huai’an, and other places [139,140]. FB1 was found to promote the value-added of normal human esophageal epithelial cells (HEECs) while decreasing the ratio of G0/G1 cells. This alteration may be due to FB1 upregulating the expression of cell cycle protein D1, which inhibits the expression of P21 and P27 [141]. Meanwhile, the expression of these three substances has been shown to be associated with esophageal cancer [142]. It has also been suggested that FB1 is a promoter or initiator of certain carcinogens, acting synergistically [143]. Recent studies have shown that FB1 functions to promote cell migration and proliferation. In HEECs, FB1 can suppress the expression of tumor suppressor genes (phosphatase and tensin homologue (PTEN) and adenomatous polyposis coli (APC)), activate the PI3K/Akt signaling pathway, and regulate cell survival. Ultimately, this leads to cell cycle dysregulation that promotes cell proliferation. HDAC have been implicated in FB1 induced carcinogenesis in HEECs [67].

Similar to pigs, FB1 also reduces cardiac contractility in humans, triggering idiopathic congestive cardiopathy (ICC). This is caused by massive blood flow into the heart and weakness of the heart [11]. Women in early pregnancy are at risk of giving birth to babies with NTDs on a long-term basis consuming foods containing high concentrations of FB1. The probability of this risk is proportional to the dose, and fetal death may occur in severe cases [10].

The organ toxicity hazard of FB1 in humans is mainly manifested from in vitro models (Table 5). FB1 has some toxic effects on human intestinal cell lines, but the toxicity is low. It has been found that at a concentration of 400 ppb, it causes peroxidation of intestinal cell lines, and elevated concentrations cause changes in mucosal concentrations as well as inflammation, but normal food contains 31.5–74.2 ppb of FB1, so it hardly causes intestinal problems in the normal daily diet [144]. In experiments using a human gastric epithelial cell line (GES-1) as an in vitro model, FB1 was found to significantly reduce cell viability, increase membrane leakage, cell death, and induce endoplasmic reticulum stress, resulting in gastrointestinal injury [75]. Recent studies have shown that the endoplasmic reticulum stress-associated PERK-CHOP signaling pathway plays a key role in FB1 damage to GES-1 [76]. This may be a new mechanism that needs further study.

FB1 also inhibits the value addition and prolonged cell cycle of the human normal hepatocyte line HL-7702, which may be related to FB1-induced changes in the expression levels of cyclins E and P21 [145]. In a human hepatocellular carcinoma cell line (HepG2), the expression levels of miR-27b and CYP1B1 protein were significantly negatively correlated, which may be a mode of liver tumor transformation [146] However, after studying FB1 levels in 271 liver cancer patients and 280 normal subjects, some scholars concluded that there is no direct association between FB1 intake and liver cancer [147]. Further studies are needed to prove the relationship between FB1 and liver cancer.

**Table 5:** Toxic effects of FB1 in human in vitro models.

Cell Type	Dosage	Duration	Effects	References
Gastric epithelial cell line (AGS) and human colon adenocarcinoma cell line (SW742).	4.5~72 mg/L	72 h	Increased levels of pro-inflammatory cytokines such as IL-1β and TNF-α and decreased IL-8 levels in gastric and colonic cell lines in a concentration-dependent manner. This effect may underlie the development or progression of inflammation and subsequent atrophy of the stomach and intestine.	[79]
Human esophageal epithelial cells (HET-IA)	1 μM or 100 μM	5 days	1 μM fumonisin B1 had no effect on the clonal growth of HET-1A, but 100 μM fumonisin B1 inhibited the clonal growth of HET-1A by 75%. Morphological observations showed that fumonisin B1 induced apoptosis of HET-1A cells.	[148]
Human oesophageal carcinoma (SNO)cells	1.25 and 10 μM or 20 μM	-	FB1 induced apoptosis in SNO cells, as evidenced by decreased survival, phosphatidylserine externalization, increased Bax protein expression, and DNA fragmentation. Caspase-dependent apoptosis started at 1.25 and 10 μM FB1, but execution at 20 μM FB1 may be mediated by a caspase-independent pathway.	[72]
Human embryonic kidney (HEK-293) cells	25 μM	48 h	HEK-293 cells are resistant to the apoptotic effects of FB1, which enhances cell survival by forming sphingosine-1-phosphate. This finding is only applicable to HEK-293 cells, and resistance to other tissues needs further study.	[149]
Human proximal tubule-derived cells (IHKE cells)	10 μM	24 h	Both caspase 3 activity and DNA fragmentation were significantly increased.	[150]
Normal human keratino- cytes (NHKc)	1 μM or 10 μM	5 days	When the concentration reached 1 μM, fumonisin B1 had no effect on the growth of keratin-forming cells, while 10 μM fumonisin B1 inhibited the clonal growth of keratin-forming cells by 42%.	[148]
NHKc	10 μM or 100 μM	4–8 days	Increased intracellular lipids in NHKc, growth inhibition at FB1 of 10 μM, and DNA fragmentation at 100 μM, all due to accumulation of sphinganine (SA).	[151]
NHKc	100 μM	2 days	The clone-forming ability of NHKc decreased to 44.5% of the control level and almost disappeared after 4 days.	[152]

### 4.3. Reproductive Toxicity

The effects of FB1 on the reproductive system mainly include reproductive failure and fetal hypoplasia in some animals [153]. FB1 reduces gonadotropin levels, inhibits granulocyte gain, and impairs normal follicle growth and oocyte survival [154]. Upon treatment of porcine cumulus oocyte complexes (COCs) with FB1 (30–40 mM), the expulsion of the first polar body was inhibited and oocyte meiotic progression was disrupted. Oocytes were arrested at the germinal vesicle breakdown (GVBD) stage. This may be because FB1 induces oxidative stress in porcine oocytes, which in turn triggers oocyte apoptosis [155]. FB1 (15 or 18 mg/kg) was administered to Syrian mice and the number of litters was significantly decreased. Mouse embryos showed stunted development after 26 h of exposure to FB1 (≥1.44 mg/kg) and after exposure to FB1 (36 mg/kg) for 2 h [156]. FB1 also reduced body mass, litter size, and feeding capacity in pregnant rats, resulting in craniosynostosis and sternal deformity in the fetus [153]. When the male rabbits were exposed to FB1 with a concentration of up to 7.5 mg/kg, the sperm reserves of testis and epididymis were reduced, sperm production was inhibited, and a potential reproductive obstacle ensued, leading to embryo death in the later stage of embryo development [156]. When exposed to 10 mg/kg FB1, mild or moderate testicular lesions and Sertoli cell degeneration were found in rabbits [105].

## 5. Perspectives

Based on the above literature, it is suggested that the toxic effects of FB1 can have a great impact on animal husbandry and also cause a certain threat to human health. In this paper, we examined the contamination of FB1 in food and feed and the mechanism of FB1 toxicity on animal and human beings. At present, the synergistic mechanism of FB1 with mycotoxins as well as other microorganisms needs further investigation. The toxic effects of FB1 on epigenetics are also a major trend in research. Further research on the toxicity mechanism of FB1 in animal and human organism can reduce the toxicity hazard and reduce the economic loss in the farming industry.

## Data Availability

Data is contained within the article.
